# Atraumatic Insufficiency Fractures of the Tarsal Bones - An Unusual Cause of Recurrent Heel Pain in a Patient with Rheumatoid Arthritis: A Case Report

**DOI:** 10.5704/MOJ.1807.012

**Published:** 2018-07

**Authors:** A Rajeev

**Affiliations:** Department of Orthopaedics, Gateshead Health NHS Foundation Trust, Gateshead, United Kingdom

**Keywords:** atraumatic, insufficiency fractures, tarsal bones, rheumatoidarthritis, methotrexate

## Abstract

The incidence of insufficiency fractures is approximately 1% in rheumatoid arthritis patients. The predisposing factors are chronic inflammation, skeletal deformities, biomechanical stresses and osteoporosis. The medications used in the treatment of rheumatoid arthritis such as Glucocorticosteroids and Methotrexate also contribute to the development of osteoporosis and insufficiency fractures. A 68-year old lady who was suffering from rheumatoid arthritis and on long term Methotrexate was seen in the outpatient clinic with recurrent episodes of heel pain. Examination revealed diffuse tenderness around the heel with full range of ankle movements but painful limitation of subtalar joint movements. Radiographic examination of the ankle showed a highly suspicious fracture of the calcaneus and confirmed on MRI as an insufficiency fracture. She was treated successfully with oral bisphosphonates and moon boot brace. She presented after two years with recurrent episodes of heel pain. The plain radiograph and MRI scan confirmed an insufficiency fracture of the talus. She was treated non-operatively with intravenous Zolendronic acid and bracing. In chronic rheumatoid arthritis patients especially on Methotrexate and Glucocorticoids a high index of suspicion of insufficiency fractures should be considered if they present with bone pain. MRI scan is the investigation of choice and is conclusive.

## Introduction

Insufficiency fractures are stress fractures due to repeated normal stress force on abnormal bone in the area of elastic resistance deficiency^1^. They are usually seen in elderly women with osteoporosis. Any condition which weakens the bone, such as rheumatoid arthritis, Paget’s disease, osteomalacia, Milkman syndrome, diabetes mellitus and long term steroid or bisphosphonate treatment can predispose to insufficiency fractures^2^. Insufficiency fracture can be easily overlooked in asymptomatic cases^1^. Although there are some reports of insufficiency fractures of tarsal bones in the literature, recurrent atraumatic insufficiency fractures of the tarsal bones in a patient with rheumatoid arthritis have not been reported. We report a rare case of atraumatic insufficiency fracture of calcaneus followed by talus in a rheumatoid arthritis patient.

## Case Report

**Fig. 1:**
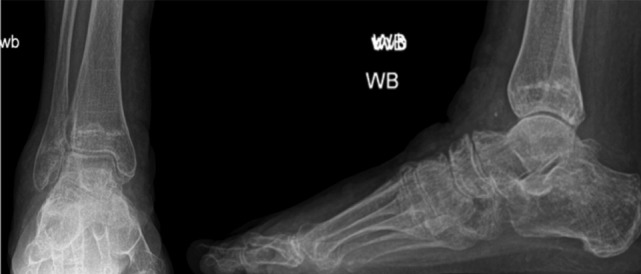
Plain weight-bearing radiographs of foot and ankle showing normal hind foot alignment.

**Fig. 2:**
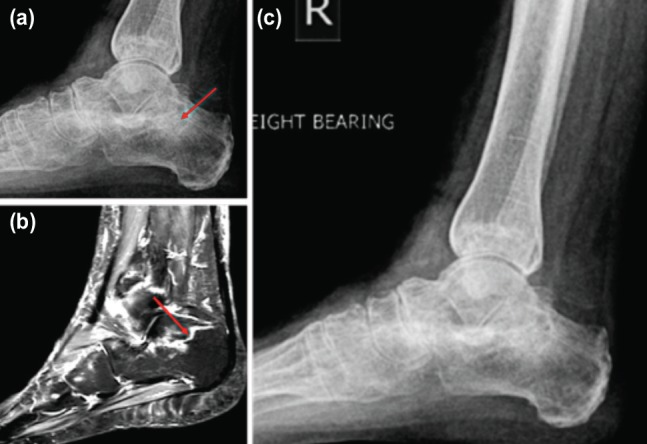
(a) Plain radiograph of the calcaneus with a suspicious fracture. (b) T2 weighted MRI images of the calcaneus showing insufficiency fracture. (c) Plain radiograph of the calcaneus showing the healed fracture.

A 68-year old lady was referred by her general practitioner to the orthopaedic clinic with complaints of right heel pain for one year. The presenting complaints started about a year back as insidious onset of heel pain which was constant aching in nature and aggravated by periods of prolonged walking and standing. There was no history of trauma. She was a known seropositive rheumatoid arthritis patient on Hydroxychloroquine, Sulphasalazine and Methotrexate for 20 years. She was under the care of a rheumatologist and had steroid injections in the right heel for plantar fasciitis in the past. On examination there was diffuse tenderness around the heel with full range of ankle movements but painful limitation of subtalar joint movements. There was no hind foot malalignment evident on weight-bearing radiographs of foot and ankle (Fig. 1). But that of the ankle revealed a sclerotic line with areas of osteolysis suspicious of a fracture of the calcaneus (Fig. 2a). An MRI scan of the ankle with T2 weighted images showed linear high signal intensity in the body of the calcaneus suggestive of an insufficiency fracture of the calcaneus (Fig. 2b). The patient was treated with moon walker boot and commenced on oral Alendronic acid 10mg on alternate days for eight weeks with Calcichew and Vitamin D tablets. The patient was advised to use the moon boot while weight bearing and remove at bed time. At the eight weeks follow-up she was completely pain free and able to fully weight bear. The radiographic examination showed the fracture had healed (Fig. 2c).

**Fig. 3:**
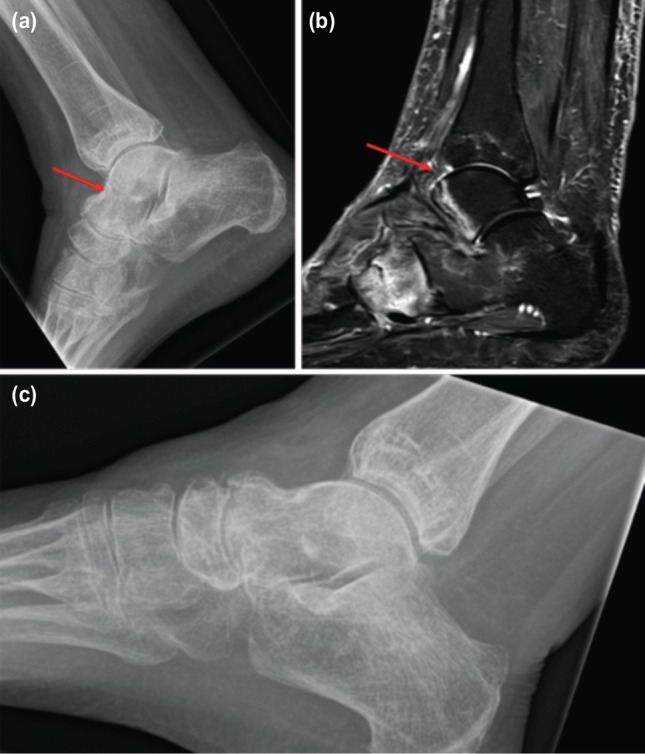
(a) Plain radiograph of the talus showing insufficiency fracture, (b) T2 weighted MRI scan of the talus showing the fracture. (c) Plain radiograph of the talus showing healed fracture.

The patient was referred back to our orthopaedic clinic after two years by her general practitioner with complaints of recurrence of right heel pain with no history of trauma. On clinical examination there was tenderness around the talus and painful restriction of ankle and subtalar movements. Radiographic examination revealed a fracture of the talus (Fig. 3a). An MRI scan of the foot and ankle showed insufficiency fracture of the head of the talus (Fig. 3b). She was treated in a moon walker boot and 5mg of intravenous Zolendronic acid as a bolus dose. She was asked to continue with oral Alendronic acid 10mg on alternate days for 12 weeks. The patient was reviewed at 12 weeks. There was no tenderness around the talus or calcaneus. A repeat radiograph showed that the fracture of the talus had healed (Fig. 3c). She has been on yearly review since.

## Discussion

Heel pain is one of the commonest complaints of patients presenting in the foot and ankle clinics. There are several causes of heel pain including local and systematic factors. Insufficiency fracture occurs due to loss of elastic resistance of the bone. It also happens due to the windlass effect of the plantar fascia and Achilles tendon which indirectly increase the load on the heel^3^. The common sites which are affected by insufficiency fractures are vertebrae, sacrum, neck and proximal third of the femur, pubic rami and sternum.

Radiographic appearance depends on the site of the fracture. Insufficiency fractures may have an appearance that depends on the stage of fracture maturity which includes sclerosis, lytic fracture line, bone expansion, exuberant callus, and osteolysis. Bone scan often shows increased uptake at the fracture site. MRI is as sensitive as bone scanning but has higher specificity. It is quite useful in finding the exact anatomic location and in distinguishing insufficiency fractures from tumours or infection.

Bisphosphonate is the treatment of choice in insufficiency fractures. It acts by normalisation of the rate of bone turnover and significant increase in bone mineral density (BMD), thereby reducing the risk of repeated fractures. In our patient we treated the insufficiency fracture of the calcaneus with oral Alendronic acid initially. Intravenous preparations of bisphosphonates such as Zoledronic acid are used in the treatment of recurrent or resistant cases especially in insufficiency fractures occurring in rheumatoid arthritis patients. When the patient presented for the second time with insufficiency fracture of the talus we gave her a bolus dose of intravenous Zoledronic Acid.

In rheumatoid arthritis foot and ankle problems are common presenting complaints. Delay in the diagnosis of insufficiency fracture is high in rheumatoid arthritis patients. The causes of insufficiency fracture in rheumatoid arthritis patients can be multifactorial. The chronic inflammation increases the risk of structural changes which may include peri-articular osteopenia, bone erosions, tendinopathies, and skeletal deformities. These may place abnormal biomechanical stresses on the feet and increase predisposition to insufficiency fractures^4^.

Patients with long-standing rheumatoid arthritis on Methotrexate medication and who present with atraumatic heel pain, a diagnosis of insufficiency fractures of the tarsal bones should be considered. Trickey *et al* in their case series of three patients with rheumatoid arthritis who presented with acute swollen ankle joints have reported that the diagnosis would be mistaken for an acute flare up of the arthritis, thereby delaying the diagnosis of insufficiency fractures. They concluded that a high index of suspicion was needed in spite of normal plain radiographs to diagnose insufficiency fractures in rheumatoid arthritis patients^5^. Our patient has been taking Methotrexate for 20 years. MRI scan is the investigation of choice especially if plain radiographs are normal. These fractures can be successfully and effectively treated with oral bisphosphonates along with bracing.

## Conflict of Interest

The authors declare no conflicts of interest.
